# The Effect of Preheating on the Mechanical Properties of AISI 1037 and AISI 304 Welded Joints Using Shielded Metal Arc Welding

**DOI:** 10.3390/ma17235780

**Published:** 2024-11-25

**Authors:** Sudarsono, Amir Arifin, Gunawan, Muhammad R. Y. Q. A. Wijaya, La Ode Ahmad Barata, Mohd Ikram Ramli

**Affiliations:** 1 Mechanical Engineering Department, Halu Oleo University, Kendari 93232, Indonesia; ahmad.barata@uho.ac.id; 2 Department of Mechanical Engineering, Sriwijaya University, Indralaya 30662, Indonesia; amir@unsri.ac.id (A.A.); gunawan@unsri.ac.id (G.); rindangs@student.unsri.ac.id (M.R.Y.Q.A.W.); 3 School of Engineering, University of Wollongong Malaysia, Glenmarie Campus, Sham Alam 40150, Malaysia; ikram.r@uow.edu.my

**Keywords:** AISI 1037, AISI 304, preheating temperature, dissimilar welded joint

## Abstract

This study explores the effect of preheating on the toughness of dissimilar welded joints between AISI 1037 and AISI 304 steels, using Shielded Metal Arc Welding (SMAW) and E309-16 electrodes. The innovation of this approach lies in assessing how preheating temperatures influence the mechanical properties of such welds. Preheating temperatures ranged from 150 °C to 300 °C, with impact testing revealing a notable increase in toughness, from 6.01 Joules at 150 °C to 19.57 Joules at 300 °C. Hardness tests indicated a maximum hardness of 313 VHN in the fusion zone and a minimum of 185 VHN in the AISI 304 area. Compared to non-preheated joints, preheating significantly enhanced impact strength and altered the fracture mode from brittle to ductile. Macrostructural and microstructural analyses with optical microscopy and SEM showcased changes in fracture surfaces and microstructural evolution, highlighting the improvement in mechanical properties due to preheating. These findings demonstrate that preheating critically enhances the toughness and overall performance of dissimilar metal welds, making it a valuable technique in industrial applications where enhanced joint toughness is crucial.

## 1. Introduction

Today, in the rapid development of the industrial era, in relation to transportation, maritime, aviation, chemical, and other fields, the use of ferrous-based materials is increasingly varied, making the joining of dissimilar metals a more common occurrence. Welding dissimilar materials is a more complicated process than that of welding similar materials because the procedure is carried out in areas where specific attributes need to be improved [1,2,3]. AISI 1037 is a carbon steel that is primarily used for its moderate strength and machinability. AISI 304 is a stainless steel that is well regarded for its corrosion resistance, making it suitable for various applications where exposure to corrosive elements is a concern. The process of joining these two materials can be classified as dissimilar welding because of the difference in their compositions [4].

The main challenge of dissimilar welding is the difference in physical and mechanical properties such as the melting point, material composition, filler metal, thermal conductivity, metallurgical structure, and corrosion resistance of the two used materials [3,5,6,7,8]. The large variety of elements in the two materials makes it difficult to avoid the formation of intermetallic compounds [3,6,9,10]. The intermetallic phase of the compound is generally hard and brittle, which causes failure in the welded joint [6].

Temperature has an important contribution in determining the behavior of steel. Steel tends to become brittle at low temperatures, resulting in decreased ductility. The “ductile to brittle transition temperature” is the temperature at which the drop in toughness takes place [11]; this phenomenon can be tested using the notched-bar impact method. Since the fracture is ductile in such a transition, the impact energy becomes quite high at higher temperatures. As the temperature is dropped, the fracture becomes more brittle, and the impact energy decreases across a limited temperature range [12]. The transition can also be detected in the fracture surfaces, exhibiting granular surfaces for completely brittle fracture and fibrous or dull surfaces for completely ductile fractures. The characteristics of both types will be present over the ductile-to-brittle transition [13].

For many materials, the transition may take place across a wide range of temperatures. However, the change can suddenly occur for pure materials at a specific temperature [14]. At transition temperatures, ferrite steels’ fracture toughness significantly changes. Steel cleaves and becomes brittle at low temperatures; however, it is ductile at high temperatures and fails as a result of plastic collapse and micro coalescence [15]. At normal temperatures, dislocation motion causes plastic deformation in metals. The amount of stress required to displace a dislocation depends on factors such as atomic bonding, crystal structure, and obstacles including solute atoms, grain borders, precipitate particles, and other dislocations. Instead, the metal will break into widely spaced cracks, and if the tension needed to realign the dislocation is too great, the failure will be brittle. Thus, depending on which mechanism calls for the lowest applied stress, a ductile failure, a crack, or a brittle failure will occur [11,14,16].

This study offers an approach to systematically investigating the influence of preheating on the mechanical properties of dissimilar welded joints between AISI 304 stainless steel and AISI 1037 carbon steel, focusing on key outcomes like weld strength, microstructural changes, and fusion integrity. Through controlled preheating temperatures, we aim to reveal how preheating can optimize thermal management, reduce residual stress, and mitigate the formation of brittle phases at the weld interface. The insights gained can provide valuable guidelines for industries using these materials, enhancing weld quality and extending the application of SMAW to broader manufacturing contexts involving dissimilar steels. Preheating, a widely utilized method in welding to reduce thermal stress and improve fusion between dissimilar materials, is expected to significantly influence weld quality and overall joint performance, especially when working with stainless steel and carbon steel [17,18].

To evaluate these effects, two primary mechanical tests will be conducted—hardness testing and impact testing. The hardness test will measure the material’s resistance to deformation, providing critical insights into the strength distribution and uniformity across the weld and the heat-affected zones [19,20]. Conversely, the impact test will assess the weld’s toughness, determining the amount of energy the material can absorb prior to fracturing. These tests are crucial for determining whether preheating improves the mechanical performance of the joint, particularly regarding its strength, toughness, and durability [21].

Additionally, fracture surface analysis will be performed on the tested specimens to visually assess the quality of the welds and the fracture behavior. This will allow for the identification of any improvements in fracture consistency, as well as the potential reduction in defects such as cracks, voids, or brittle failures. By integrating the results from these mechanical tests with fracture surface observations, this study aims to provide a comprehensive understanding of how preheating affects the performance and reliability of dissimilar metal welds [22].

Shielded Metal Arc Welding (SMAW), also known as stick welding, is a manual arc welding process where a consumable electrode, coated in flux, is used to join metals by producing a weld. The flux coating on the electrode serves multiple purposes—it shields the molten weld pool from atmospheric contamination, stabilizes the arc, and adds alloying elements to the weld. Recent developments in welding technologies have continued to enhance the capabilities of SMAW, making it even more relevant in today’s manufacturing and construction sectors, despite the emergence of more advanced processes [23,24].

One of the reasons that SMAW remains widely used is its versatility and adaptability. It is compatible with a wide range of materials, including carbon steels, stainless steels, and alloyed metals, which makes it a popular choice for various industries. In recent years, improvements in consumable electrode design have expanded its applicability to more complex materials, including high-strength steels and corrosion-resistant alloys. This has allowed SMAW to maintain its relevance even in industries that demand higher-quality and stronger welds, such as in pressure vessel construction, shipbuilding, and pipeline welding [2,25].

Furthermore, advancements in portable welding power sources have enhanced the portability of SMAW equipment, making it a preferred option for fieldwork and on-site repairs. Modern inverter-based welding machines are more compact, energy efficient, and capable of delivering more stable arcs, even in difficult welding positions. This has improved weld quality and consistency in outdoor environments where fluctuating conditions, such as wind or temperature, can otherwise compromise weld integrity.

SMAW’s ease of use is another factor that has contributed to its sustained popularity. Recent training programs and certification efforts have focused on enhancing welders’ skills and knowledge, ensuring that even as technology advances the welding process remains accessible to a wide range of technicians and workers. The development of better safety equipment and personal protective gear has also made SMAW safer and more ergonomic for operators, reducing the risks associated with manual welding processes.

In comparison to newer welding technologies like Gas Tungsten Arc Welding (GTAW) or Gas Metal Arc Welding (GMAW), SMAW continues to offer cost-effectiveness. The process does not require external shielding gasses, and the equipment and consumables are relatively inexpensive, which is advantageous for smaller operations or field applications with limited resources. This economic advantage, combined with recent developments in electrode technology, welding machines, and safety measures, has allowed SMAW to remain competitive in a market where automation and robotics are increasingly prevalent.

**Figure 1:**
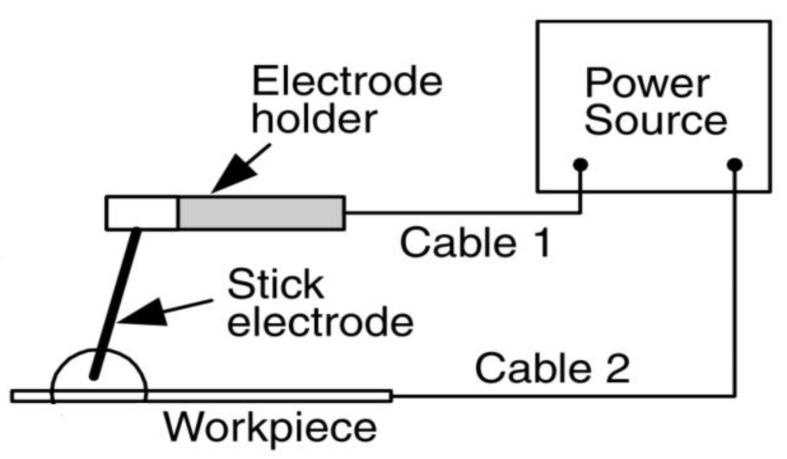
SMAW schematic [23].

In summary, while more sophisticated and automated welding methods have been developed, SMAW continues to be a vital process due to ongoing improvements in equipment, electrode design, and welder training. These advancements ensure that SMAW remains a reliable, versatile, and cost-effective option for industries requiring durable welds across a variety of materials and conditions. The SMAW schematic can be seen in Figure 1.

In Shielded Metal Arc Welding (SMAW), several parameters are crucial for determining the quality of the welding joint [2,26,27]. The welding current must be properly set to ensure adequate penetration and fusion; too high a current can led to excessive spatter and burn-through, while too low a current can cause incomplete fusion and weak joints. The arc length, or the distance between the electrode tip and the workpiece, affects the stability of the arc and the quality of the weld, with a consistent, optimal arc length producing a uniform bead. The electrode angle influences the direction and stability of the arc, as well as the shape and penetration of the weld bead; incorrect angles can lead to defects such as undercutting or a lack of fusion. The travel speed at which the electrode is moved along the joint affects bead shape and penetration; too fast a speed can result in a narrow, shallow weld, while too slow a speed can produce a wide, convex bead with excessive reinforcement and possible slag inclusion. The correct choice of electrode type and size is essential for achieving the desired weld properties, including strength, toughness, and resistance to cracking. The choice of polarity, whether direct current (DC) or alternating current (AC), affects penetration, bead shape, and arc stability, making it important to select the appropriate polarity for the electrode and base material. The proper cleaning and preparation of the base material, including the removal of rust, paint, oil, and other contaminants, are vital to prevent defects such as porosity and inclusions. Additionally, the total heat input, which is a function of current, voltage, and travel speed, influences the metallurgical properties of the weld and the heat-affected zone (HAZ), requiring careful control to avoid issues such as distortion, cracking, and changes in mechanical properties. Lastly, environmental conditions like wind, humidity, and temperature can impact the welding process; for instance, excessive wind can disrupt the shielding gas, leading to porosity, while extreme temperatures can affect the welder’s ability to control the weld pool. By carefully monitoring and controlling these parameters, welders can produce high-quality welds with the desired mechanical properties and minimal defects.

## 2. Materials and Methods

Two types of steel were selected for this study—AISI 304 stainless steel and AISI 1037 carbon steel. Each was used in plate form with dimensions of 500 mm × 150 mm × 10 mm. The nominal chemical compositions of these materials prior to welding are detailed in Table 1. The careful selection of materials ensures an accurate representation of typical industrial welding scenarios. The plates were joined using the Shielded Metal Arc Welding (SMAW) technique. An E309-16 electrode, compatible with the dissimilar metal welding of stainless and carbon steels, was employed. The welding operation utilized a current of 115 Amperes. For preparation, a single V-Groove joint configuration was applied to facilitate the proper penetration and fusion of the weld metal across the joint interface. To mitigate the risk of thermal stresses and improve the weld quality, preheating was applied uniformly across the joint area. The preheating temperatures varied at 150 °C, 200 °C, 250 °C, and 300 °C, with each temperature being maintained for a duration of 15 min prior to the commencement of welding based on AWS D1.1. Preheating temperatures around 150 °C to 200 °C were used for low-carbon and structural steels to control hydrogen-induced cracking by reducing diffusible hydrogen. For thicker sections or high-strength alloys, a higher preheating temperature may be applied depending on hardness control needs or welding codes. Moreover, a 15 min hold time allows for temperature consistency across the joint area before welding begins, which is particularly useful in high-restraint or low-hardenability applications. This treatment helps in reducing the cooling rate, thereby minimizing the risk of crack formation in the welded zones. Preheating in this experiment was carried out using a torch.

Post welding, specimens were fabricated from the welded plates to meet the requirements of the JIS Z2242 standard for impact testing. The standard specifications for the impact test specimens are as follows: a length of 55 mm, a width of 10 mm, and a height of 10 mm. The notch type should be a V-notch with a 45-degree angle, a depth of 2 mm, and a root radius of 0.25 mm. Samples for impact testing were prepared for 5 repetitions. The welding parameters for this experiment can be seen in Table 1.

**Figure 2:**
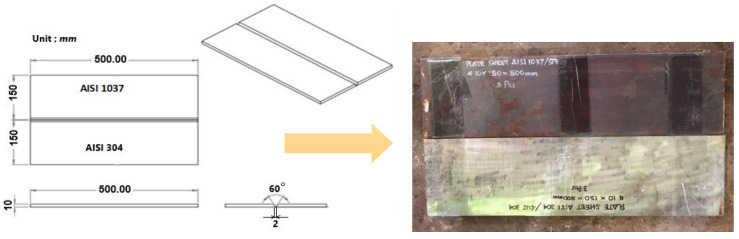
SMAW of the metal specimen.

Tests were conducted using a Charpy impact tester, specifically the CL-30 model, under ambient conditions. This analysis was pivotal in assessing the toughness and energy absorption characteristics of the welded joints. The influence of welding and heat exposure on the hardness profile was evaluated using the Vickers hardness method. Measurements were performed with a VKH-2E Vickers hardness tester across sections of the welded joint. This analysis provided a comprehensive profile of the hardness changes induced by the thermal welding cycle. The microstructural properties of the welded joints were characterized through metallographic techniques. Different etchants were applied based on the materials, i.e., 3% Nital for AISI 1037 carbon steel and Aqua Regia for the weld zone and AISI 304 stainless steel. These etchants reveal microstructural features by selectively corroding the surfaces. Observations were conducted using a Keyence VH-Z450 optical microscope by Keyence Corporation, Osaka, Japan, as well as a high-resolution Field Emission Scanning Electron Microscope (FESEM)—Thermo Scientific Quattro S model—to capture detailed images of the microstructure, aiding in understanding the phase transformations and grain structure modifications post welding. Figure 2 shows the size of the welded specimen.

## 3. Results and Discussion

X-ray fluorescence (XRF) was carried out to determine the chemical composition in three zones—AISI 1037 carbon steel base metal, AISI 304 stainless steel base metal, and the fusion zone.

The base metal composition of AISI 1037 carbon steel contains five elements, as shown in Table 2. The main alloying element obtained is Mn (0.631%). The Mn content of carbon steel can increase the hardness value of the material. Some elements in the 304 stainless steel base metal zone contain percentages detected using the XRF tool. The main alloying element obtained is Cr (18.07%). The Cr content significantly influences the corrosion resistance properties of stainless steel. In addition, Cr also increases toughness and the ability to be hardened. The second main alloying element is Ni (8.08%). Nickel itself increases toughness, increases corrosion resistance, and reduces stress corrosion cracking [28]. As concerns the XRF results obtained in the fusion zone, the percentage of Cr detected is 23.64%—the most significant element in the alloy composition. On the other hand, the minimum alloying element detected is Sb, at 11.59%. The high concentration of chromium alloys is believed to come from the incoming alloying elements due to the filler (E309-16) used during welding.

Impact testing was carried out to investigate the impact strength or toughness of the welded joints by calculating the amount of impact energy absorbed during the test process until a fracture occurs. The toughness of a material represents the material’s ability to withstand fractures caused by the presence of a notch or a stress concentration. Temperature changes play an essential role in determining the toughness value of steel [29]. In this work, impact testing was carried out at four preheating treatment conditions—150 °C, 200 °C, 250 °C, and 300 °C—as well as without heat treatment, before welding was performed.

### 3.1. Impact Strength of Weld Joints

**Figure 3:**
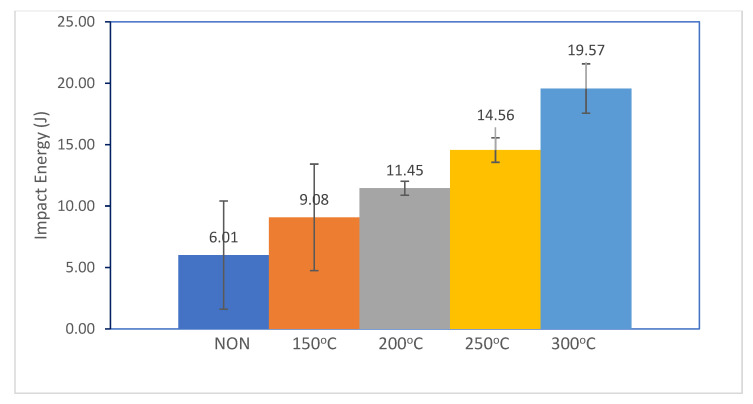
Impact test results for SS 304 and AISI 1037 steel joining.

The impact testing results obtained using the Charpy method showed that SS304 and AISI 1037 welded joints increased with preheating temperature up to 300 °C, as shown in Figure 3. The maximum impact value was achieved at a temperature of 300 (19.57 Joules), while the minimum value was achieved in the sample that was not heat treated (6.01 Joules). The impact test results clearly demonstrate the correlation between preheating temperature and the toughness of the SS 304 and AISI 1037 steel joints. As the preheating temperature increases, the impact energy absorbed by the material also increases, indicating enhanced toughness. This can be explained through the lens of the ductile-to-brittle transition (DBTT) diagram and the concept of impact strength.

Impact strength refers to a material’s ability to absorb energy upon sudden impact. A material with a higher impact strength can withstand greater impact forces without experiencing significant damage or deformation. The ductile-to-brittle transition diagram illustrates the relationship between a material’s temperature and its propensity for ductile or brittle behaviors. At higher temperatures, materials tend to be ductile; they deform plastically before breaking, absorbing a significant amount of impact energy. On the other hand, at lower temperatures, materials become brittle; they fracture with minimal deformation, absorbing minimal energy. The impact test results align with the concept of the DBTT diagram. As the preheating temperature increases, the material experiences a shift towards the ductile region of its DBTT. This is reflected in the higher impact energy values observed at higher preheating temperatures.

With non-preheated conditions, the low impact energy in the non-preheated state suggests that the material is operating in a brittle regime, near its DBTT. With preheated conditions (150 °C, 200 °C, and 250 °C), as the preheating temperature rises, the impact energy increases, indicating an increase in ductility. The material is moving towards the ductile region of its DBTT. The highest impact energy observed at 300 °C signifies that the material is well within its ductile region, having moved significantly away from its DBTT. This state corresponds to a significantly higher toughness. The impact test results provide evidence that increasing the preheating temperature enhances the toughness of the SS 304 and AISI 1037 steel joints. This is directly attributed to the materials transitioning towards more ductile states, as depicted in the DBTT diagram. By elevating the preheating temperature, the materials gain greater resistance to impact forces, leading to increased impact strength and overall toughness.

### 3.2. Hardness Measurements

**Figure 4:**
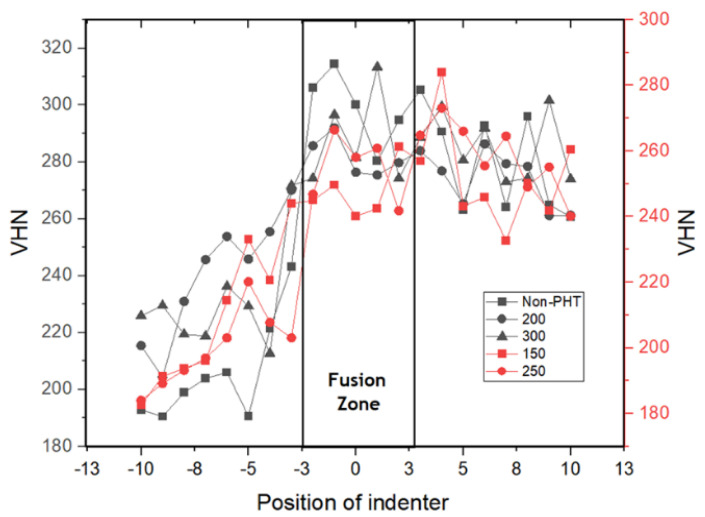
Hardness distribution of weld sample joints with preheating temperatures (non−PHT, 200 °C, 300 °C, 150 °C, and 250 °C).

An analysis of the distribution of hardness values at welded joints was carried out using the Vickers hardness method. Figure 4 shows the Vickers hardness number (VHN) distribution across different zones of a weld sample. The weld samples were preheated to various temperatures as follows: no preheating (Non-PHT), 150 °C, 200 °C, 250 °C, and 300 °C. The x-axis represents the position of the indenter relative to the weld centerline, with the fusion zone marked.

The fusion zone generally exhibits a lower hardness compared to the heat-affected zones (HAZs). This is due to the melting and rapid solidification, resulting in coarse grains and a less dense microstructure, which, in turn, leads to a reduced hardness. Variations within the fusion zone itself are likely due to cooling rate variations and solute segregation. The exact composition of the fusion zone will be a blend of the SS304 and AISI 1037, with the potential formation of intermetallic phases depending on the welding process parameters. Heat-affected zones (HAZs), as seen in Figure 4, show an increase in hardness relative to the respective base metals. The extent of this increase differs significantly depending on the base material.

For the SS304 HAZ, the increase in hardness might be less pronounced compared to the AISI 1037 HAZ. Austenitic stainless steels (like SS304) are less susceptible to significant hardness changes due to heat input because they do not undergo martensitic transformations. Any hardness increase would likely be due to strain hardening from the welding thermal cycle. In the AISI 1037 HAZ, the increase in hardness is more significant. Low-carbon steels (like AISI 1037) are more prone to microstructural changes upon heating and cooling during welding. The hardness increase is linked to the formation of martensite or bainite due to rapid cooling, both of which are significantly harder than the ferrite–pearlite microstructure of the base metal. Moreover, the increase in preheating temperature generally reduces the peak hardness in the HAZ for both base materials. This is because higher preheating temperatures lead to slower cooling rates, suppressing the formation of hard martensitic phases in AISI 1037 and reducing strain hardening in SS304.

### 3.3. Surface Morphology of Weld Joint

**Figure 5:**
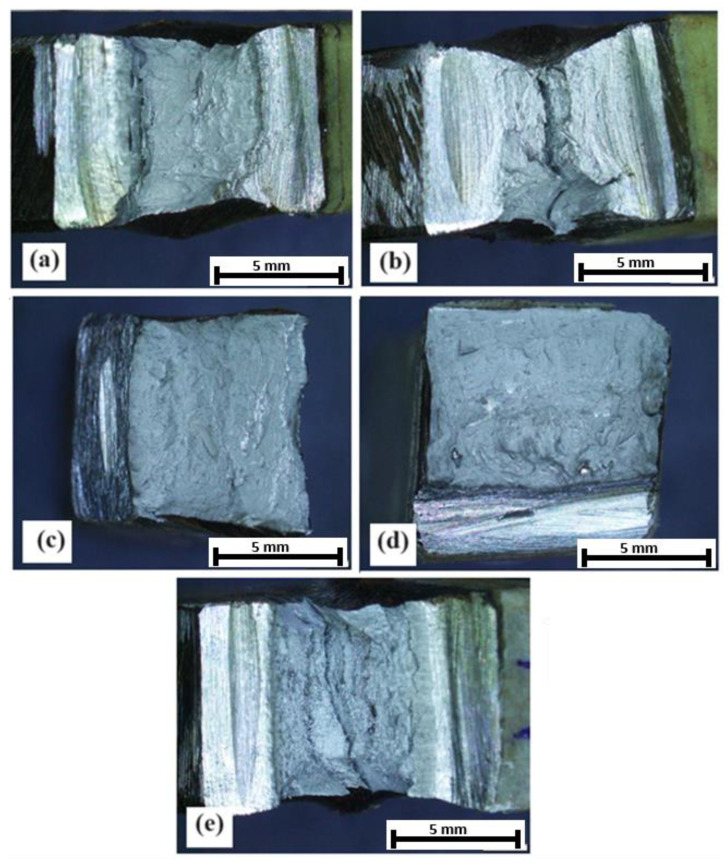
Surface morphology of the welded joint after Charpy impact test. (**a**) Without preheating; (**b**) at 150 °C; (**c**) at 200 °C; (**d**) at 250 °C; and (**e**) at 300 °C.

Figure 5 shows the morphology of welded joint surfaces on impact test specimens. Figure 5a shows the sample that was not reheated, whereby the fracture surface exhibits a brittle fracture pattern, characterized by a rough, uneven surface with a lack of significant ductility. This indicates that the weld metal was unable to effectively absorb the impact energy.

Figure 5b shows the sample that was preheated at 150 °C, whereby the fracture surface displays a transition from a brittle to a ductile fracture. Some ductile tearing is evident, indicating that the weld metal has gained some toughness due to preheating. Figure 5c shows the sample that was preheated at 200 °C, whereby the fracture surface shows more pronounced ductile tearing, implying that preheating at this temperature further enhances the weld metal’s toughness. The fracture surfaces become progressively smoother and display evidence of ductile tearing, indicating that the weld metal is becoming more resilient to impact forces. This positive impact of preheating can be attributed to several metallurgical factors. Firstly, the reduced cooling rate due to preheating promotes the formation of a finer grain structure, which is inherently tougher. Secondly, preheating reduces internal stresses, allowing the material to deform more readily under impact. Figure 5d (the sample preheated at 250 °C) shows that the weld exhibits a substantial increase in ductility. The fracture surface is characterized by significant ductile tearing and a smoother appearance. In Figure 5e, the weld displays the highest ductility, evidenced by a relatively smooth and even fracture surface with extensive ductile tearing. This indicates that preheating at these temperatures effectively mitigates the tendency for brittle fracture, making the welded joint much more resilient to impact loading. Preheating significantly enhances the weld metal’s toughness, allowing it to more effectively absorb and dissipate impact energy.

The surface analysis highlights the crucial role of preheating in influencing the fracture behavior of welded joints. By controlling the cooling rate and reducing the internal stresses, preheating promotes the formation of a finer, more ductile microstructure, leading to improved toughness. The visual evidence presented in these images underscores the importance of proper heat treatment practices in ensuring the safety and reliability of welded structures.

The surface analysis of the fractured welded joints provides valuable information for material scientists, engineers, and manufacturers. These data aid in optimizing preheating protocols for specific materials and applications, ensuring that welds possess the desired impact toughness for safe and reliable service. By understanding the impact of preheating on the fracture behavior of welded joints, we can create more durable and resilient structures that can withstand demanding environments and impact loads.

### 3.4. Microstructure Observation

Metallographic observations were conducted to investigate the evolution of the microstructures that occurred in welded joints. This metallographic analysis uses the Keyence VH-Z450 microscope by Keyence Corporation, Osaka, Japan.

**Figure 6:**
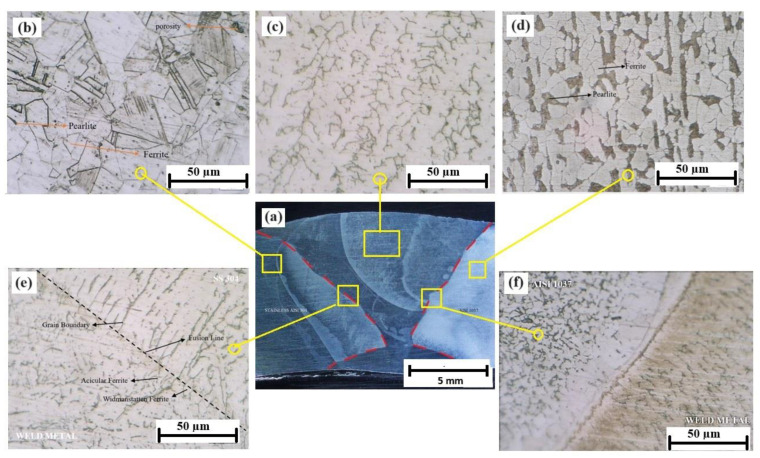
Microstructural evolution in AISI 1037 and SS AISI304 welded joints. (**a**) Macrography of the welded joints, (**b**) AISI 304 base metal zone, (**c**) fusion zone, (**d**) AISI 1037 base metal zone, (**e**) weld metal and SS 304 fusion line, and (**f**) weld metal and AISI 1037 fusion line.

Figure 6 shows the evolution of the microstructure experienced by AISI 1037 and SS AISI 304 welded joints. Figure 6a shows the welded joints of the fusion zones, heat-affected zones, and base zones of AISI 1037 and SS AISI 304. Figure 6b shows the typical microstructure of austenitic stainless steel, while Figure 6d shows a carbon steel microstructure comprising pearlite and ferrite phases.

**Figure 7:**
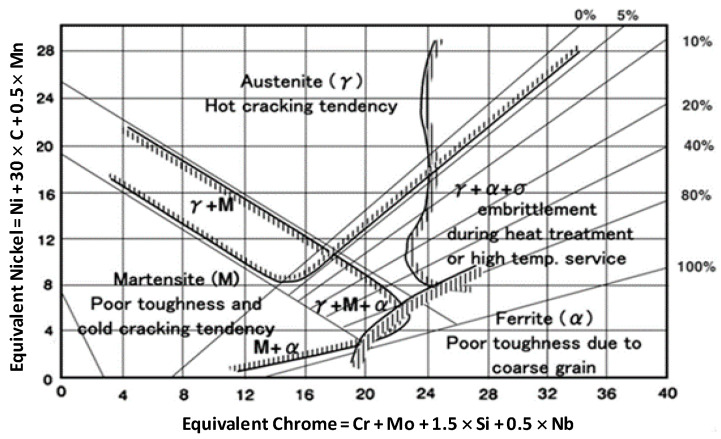
Phase prediction utilizing the Schaeffler diagram [32].

Figure 6e,f illustrate the fusion lines that mark the boundaries between the fusion zone and the base metal zone, highlighting the epitaxial growth of weld metal near the fusion line. The existing base metal grains at the fusion line serve as substrates for the nucleation process. The molten metal in the weld pool is in close contact with these substrate grains, fully wetting them. As a result, the molten metal nucleates easily on the grains of the substrate. Epitaxial growth in materials with a face-centered cubic or body-centered cubic crystal structure forms columnar dendrites oriented in the <100> direction [23]. Columnar dendrites and the fusion line of SS 304 and the weld metal at a preheating temperature of 250 °C can be seen in Figure 7.

The Schaeffler diagram is often used to predict phase formation in the fusion zone after the welding process [30]; this is closely related to phase formation in the fusion zone [31,32,33]. With carbon concentrations as low as 0.12%, the Schaeffler diagram is a suitable method for determining the weld composition of austenitic Cr-Ni steels. The percentage of the total weight is used to denote each composition concentration. Figure 7 demonstrates the phases that will occur based on the interaction of the alloy composition, as determined by Ni_eq_ and Cr_eq_ calculations.

**Figure 8:**
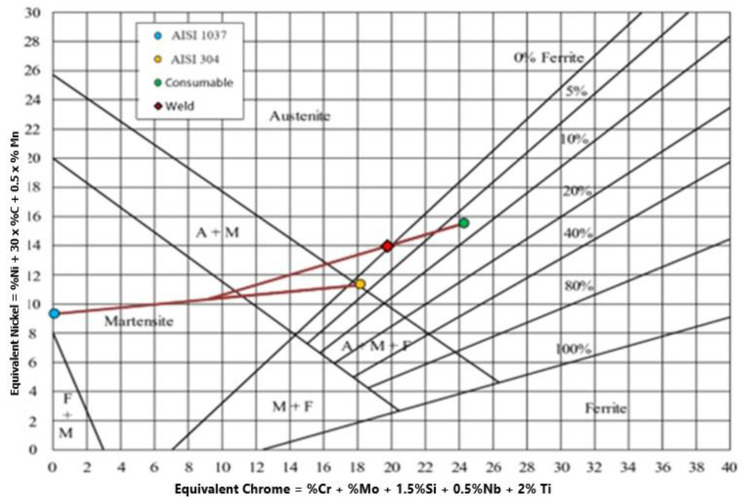
Prediction of the fusion zone between AISI 1037 steel and AISI 304 austenitic stainless steel using an E309 electrode.

Figure 8 shows the phase prediction optimal parameter for a 304 austenitic stainless steel and AISI 4340 steel weld connection using E309. The welding condition dilution ratio in this operation is around 15%. As illustrated in Figure 8, the Schaeffler diagram demonstrates that using E309 electrodes to weld AISI 1037 and AISI 304 exhibits austenite microstructures. In this state, hot cracking is frequently a problem in the fusion zone with austenite microstructures. Welds made of stainless steel experience hot cracking due to low-melting eutectics like S and P, as well as alloy elements like Ti and Nb [34]. In addition, a number of additional conditions, such as residual stress mixed with stress concentrations, including weld defects, can result in brittle failure.

**Figure 9:**
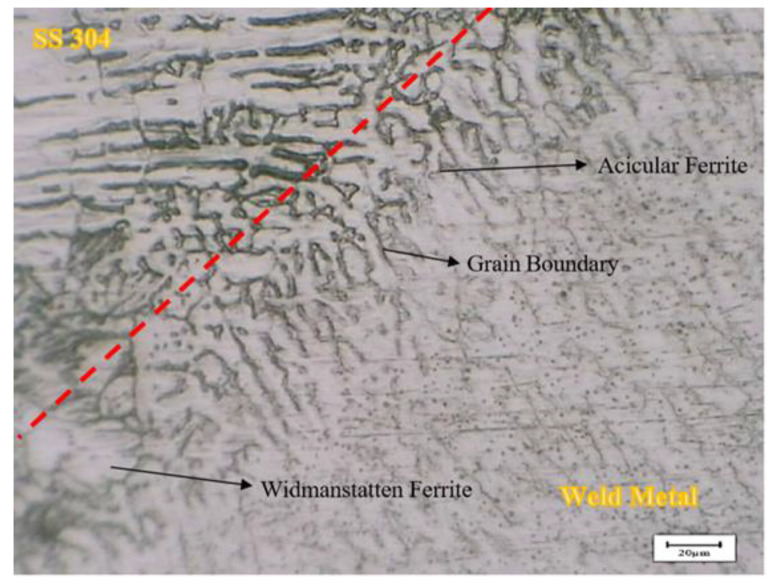
Fusion line between SS 304 and the weld metal at a preheating temperature of 250 °C.

Figure 9 shows a partially melted zone (PMZ) [35] in the form of grain boundary thickening along the fusion line. The area between the 100% melting in the fusion zone and the 100% solid zone of the base metal is the PMZ, which is a subset of the heat-affected zone (HAZ).

The microstructural analysis of the SS 304 weld zone, preheated to 250 °C, revealed distinct features that highlight the impact of preheating and welding on the weld microstructure. The base metal exhibits a typical austenitic microstructure with a fine-grained, random arrangement of austenite grains. The fusion line, marking the interface between the base metal and the weld metal, is clearly visible. At the fusion line, a remarkable feature is the presence of dendrites extending from each grain point in a single direction, varying from grain to grain. This is indicative of directional solidification as the weld pool cools, with the dendrites growing preferentially in the direction of heat dissipation.

Additionally, epitaxial growth is observed near the fusion line. This phenomenon, where crystals grow with a specific orientation relative to an existing crystal, is evident in both austenite and ferrite phases across the fusion line; this indicates that the crystallographic orientation of the base metal can influence the growth of the weld metal, leading to a degree of continuity in the microstructure across the interface.

The presence of both acicular and Widmanstatten ferrite phases in the weld metal is a direct consequence of the 250 °C preheating temperature and the heat input during welding. The preheating influences the cooling rate of the weld, promoting the formation of Widmanstatten ferrite alongside acicular ferrite. However, the heat input further shapes the microstructure.

The heat input during welding significantly influenced the grain size and phase formation in the fusion zone and the heat-affected zone (HAZ). The HAZ, where the base metal experiences elevated temperatures but does not melt, can be further subdivided into the normalized zone and the overheated zone. The normalized zone, heated to just above A3 (austenite transformation temperature), experiences grain refinement, while the overheated zone, heated significantly above A3, undergoes grain coarsening and can exhibit partially oriented Widmanstätten ferrite patterns.

The presence of these distinct ferrite phases and the observed epitaxial growth have significant implications for the weld’s mechanical properties and corrosion resistance. Acicular ferrite contributes to toughness and impact resistance, while Widmanstatten ferrite, depending on its morphology and distribution, can affect the weld’s corrosion resistance. The epitaxial growth, while contributing to a degree of continuity in the microstructure, might also influence the stress distribution and crack propagation behavior.

The 250 °C preheating temperature serves a crucial purpose in reducing thermal stresses and mitigating the risk of cracking in the weld. The preheating effectively manages the thermal gradients during welding, thereby minimizing the likelihood of hot cracking. This is particularly important for materials like SS 304, which are prone to cracking due to their high thermal conductivity.

## 4. Conclusions

This study investigated the microstructural characteristics and mechanical properties of dissimilar weld joints between AISI 1037 carbon steel and AISI 304 stainless steel using the Shielded Metal Arc Welding (SMAW) method with preheating. Preheating temperatures between 150 °C and 300 °C resulted in a significant enhancement in toughness, with impact testing showing an increase from 6.01 Joules at 150 °C to 19.57 Joules at 300 °C. The analysis focused on the influence of the preheating temperature and heat input on the weld microstructure and its implications for weld performance, specifically evaluating impact strength through Charpy impact testing.

The results emphasized that preheating markedly improves the mechanical properties of the weld joints. Hardness evaluations revealed a peak measurement of 313 VHN in the fusion zone and a minimum of 185 VHN in the AISI 304 region. Furthermore, the preheating process effectively altered the fracture mode from brittle to ductile behavior, corroborating the increase in impact strength observed with preheating temperatures up to 300 °C. This improvement is attributed to the influence of preheating on the weld microstructure, facilitating the formation of more ductile phases and reducing the likelihood of brittle fracture.

Microscopic analysis revealed epitaxial growth at the fusion line, showcasing crystallographic continuity between the austenite and ferrite phases, which is likely to affect mechanical properties and stress distribution in the weld zone. This study also identified that different preheating temperatures and heat inputs significantly influenced the formation of acicular and Widmanstätten ferrite phases, with acicular ferrite contributing to the toughness and the morphology of Widmanstätten ferrite, potentially impacting corrosion resistance.

The heat-affected zone (HAZ) was found to consist of a normalized zone with grain refinement, contrasting with an overheated zone exhibiting significant grain coarsening and partially oriented Widmanstätten ferrite structures. Overall, these findings highlight the critical role of preheating in dissimilar welding applications, demonstrating that appropriate preheating temperatures can substantially improve the microstructure and mechanical properties of welded joints, leading to enhanced toughness and a reduced susceptibility to brittle fracture.

## Figures and Tables

**Table 1:** Welding process parameters.

Parameter	Description
Joint Type	Single V-Groove butt joint
Welding Position	1G
Electrode	AWS E309-16
Welding Current	115A
Number of Layers	Six layers
Cleaning Method	Grinding and wire brush
Preheating Temperature	150 °C to 200 °C

**Table 2:** Base metal and weld metal chemical composition (by weight %).

Alloy	AISI 1037	SS 304	Fusion Zone
Cr		18.07	23.64
Ni		8.08	11.59
Mn	0.63	1.680	1.46
Si			0.35
Mo		0.09	0.10
Sb			0.04
Nb	0.03	0.03	
P	0.04		0.057
Ti	0.08		
Fe	98.2	71.28	61.92

## Data Availability

The original contributions presented in the study are included in the article, further inquiries can be directed to the corresponding author.
